# Fistula from left main coronary artery to pulmonary trunk

**DOI:** 10.1007/s12471-020-01406-0

**Published:** 2020-03-10

**Authors:** N. Papakonstantinou, N. Miaris, K. Argyrakis, S. Mitsiadis, A. Dimopoulos, G. Gavrielatos, N. Patsourakos, N. Kasinos, A. Theodosis-Georgilas, E. Pisimisis

**Affiliations:** Cardiology Department, “Tzaneio” General Hospital of Piraeus, 18536 Piraeus, Greece

An 81-year-old male patient with past medical history of arterial hypertension and diabetes mellitus presented to our emergency department with New York Heart Association class IV heart failure. His electrocardiogram showed sinus rhythm with right bundle branch block and left anterior hemiblock, while echocardiography revealed left ventricular dilation with impaired systolic function (estimated ejection fraction of 30%) and apical, posterior and inferior wall akinesia. Invasive coronary angiography was performed (Fig. 1a, b and online video 1a, 1b).

**Fig. 1:**
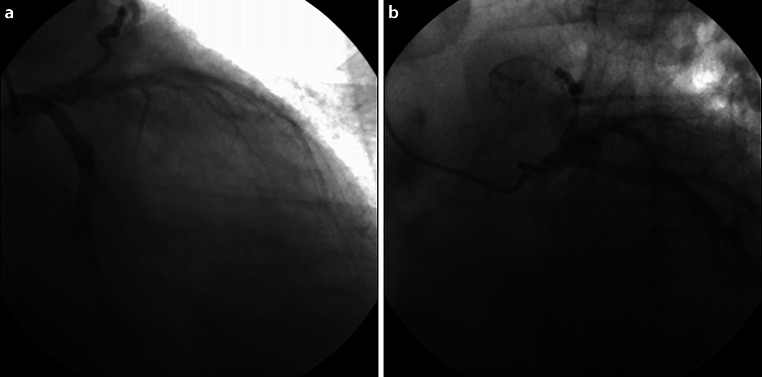
Invasive coronary angiography. **a** Right anterior oblique (RAO) caudal view; **b** Left anterior oblique (LAO) caudal view

What is the diagnosis?

## Answer

You will find the answer elsewhere in this issue.

## Caption Electronic Supplementary Material

A fistula arising from the left main coronary artery is revealed.

*Video 1b.* Left anterior oblique (LAO) caudal view of invasive coronary angiography.

